# Competencies of military nurse managers: A scoping review and unifying framework

**DOI:** 10.1111/jonm.13068

**Published:** 2020-07-12

**Authors:** Huijuan Ma, Theodora Nomusa Chihava, Jingjing Fu, Suofei Zhang, Lei Lei, Jing Tan, Li Lin, Yu Luo

**Affiliations:** ^1^ School of Nursing Third Military Medical University Chongqing China

**Keywords:** competencies, military, nurse manager, scoping review

## Abstract

**Aim(s):**

To identify competencies of military nurse managers and develop a unifying framework of military nurse managers’ competencies.

**Background:**

Military nurse managers shoulder multiple responsibilities because of duality roles, and they should possess competencies that enable them to manage human and material resources during peacetime and wartime. Therefore, nursing management within military context is demanding, such that a comprehensive understanding of their competencies is needed for effective military nursing management. Although relevant studies have focused on different military branches and different levels of managers, there is no standard evaluation framework.

**Evaluation:**

A scoping review of studies focusing on competencies of military nurse managers from seven databases was carried out.

**Key issues:**

Nine studies were included in this review, and a framework consisting of six domains of military nurse managers’ competencies was identified: clinical expertise, role model, leadership competencies, human competencies, financial competencies and deployment competencies.

**Conclusion:**

Existing knowledge of competencies of military nurse managers is limited, and a comprehensive understanding of this topic can provide direction for future work.

**Implications for Nursing Management:**

Military nurse managers play substantial roles within the military nursing context. A unifying framework can facilitate personnel recruitment and competency measurement, as well as training protocol development.

## 1 INTRODUCTION

An increase in health care demands and the ever‐changing health care environment has resulted in nurse managers shouldering most of the responsibility of increasing patient and staff satisfaction (Emmons, 2018). Nurse managers at the executive level are in charge of clinical nursing services, strategic planning, administration as well as clinical leadership, while the head nurses or first‐line nurse managers are responsible for patient care activities that occur 24 hr a day in their units (Chase, 2010; Duffield, Gardner, Doubrovsky, & Wise, 2019; Gunawan & Aungsuroch, 2017). Notably, nursing managers are responsible for maintaining the link between an institution's administrative mission and the nurses who provide nursing care in the clinical unit, as well as being in charge of efficient patient care activities by ensuring that subordinate nurses are qualified for the tasks allocated to them. This is one of the reasons why their roles are considered most complex in health care institutions (Chase, 2010). Additionally, the leadership style of nurse managers has huge influences on staff members’ turnover and the quality of patient care they deliver; so, they should have the necessary competencies to ensure the smooth running of their units (Pishgooie, Atashzadeh‐Shoorideh, Falcó‐Pegueroles, & Lotfi, 2019; Saleh et al., 2018).

Historically, military nurses were indispensable during wars, disasters and United Nations (UN) peacekeeping missions as evidenced by Florence Nightingale when she cared for wounded soldiers during the Crimean War. It is still common practice for military nurses to be deployed in response to natural disasters or epidemics to save human life. The scope of practice for a military nurse is twofold as a soldier and a professional nurse practising in the fluctuating military health care environment and meeting demanding military operational requirements which differs from the nursing environment of civilian nurses (Griffiths & Jasper, 2008; Lundberg, Kjellström, & Sandman, 2019). When deployed in a disaster area or a combat zone, military nurses deliver medical tasks in an austere environment. One common challenge military nurses face during deployment is to adapt to the dangerous environment which results in fear of the unknown (Rivers, 2016; Rivers & Gordon, 2017). Military nurses also face ethical dilemmas when facing multiple patients and shortage of supplies (Almonte, 2009). This is further confounded by the fact that, at times, military nurse officers lacking experience during deployment are elevated to head nurses of medical missions because of their military rank (Beaumont & Allan, 2012). Thus, the management competencies of military nurses differ slightly from those of civilian nurses because of the versatile environment causing physical and psychological strains. Other expectations of a military nurse manager include ensuring the quality and safety of patients and personnel in a complex working environment and acting as a role model who has a positive impact on junior nurses and encourages the team to overcome challenges (Barr, Ferro, & Prion, 2019).

To ensure high‐quality patient care, many scholars and institutions have studied and explored leadership and management competencies over the past decades. Among the published assessment tools of competencies of nurse managers, the Chase Nurse Manager Competency Instrument (NMCI) is a valid and reliable measurement scale of nurse managers’ competencies, which was developed by Dr. Chase in 1994 and further validated in 2010, based on the American Organization of Nurse Executive (AONE) Leadership Collaborative Framework and Katz's conceptual framework (Chase, 1994, 2010, 2012; Katz, 1955). The Chase competencies include Chase Technical, Chase Human, Chase Conceptual, Chase Leadership and Chase Financial Management. Further exploration of managerial competencies of first‐line nurse managers through concept analysis by Gunawan and Aungsuroch (2017) revealed attributes of managerial competencies including self‐development, planning, organising, budgeting, leadership, legal and ethical issue management and health care delivery.

However, the competencies identified above do not adequately address the demands for military nurse managers acting as both a nurse and a soldier. As a professional leader in the military, they should meet the military standards such as loyalty, discipline and obeying superior commands no matter the consequences. According to the Army Leader Requirements model, army leaders should have eight core leadership competencies including leading others, extending influence beyond the chain of command, leading by example, communication, creating a positive environment, preparing self, developing others and getting results (Funari, Gentzler, Wyssling, & Schoneboom, 2011). Additionally, the Uniformed Services University (USU) develops uniformed medical officers through the Department of Military and Emergency Medicine (MEM) by synthesizing three core competencies, which are leadership, military skills and medical skills (O'Connor, Grunberg, Kellermann, & Schoomaker, 2015). There are several studies focusing on competencies of military nurse managers; however, most of them focus on either single branch of military (army or navy), or single level of position (executive level, military nurse officer or head nurse). Thus, there is no unifying framework of military nurse managers’ competencies that can be used as an evaluation standard. To develop this unifying framework that is designed for military nurse managers with dual roles in the military health services, this paper fully and systematically evaluated existing findings of military nurse managers’ competencies to generate a scoping review comprising competencies of military nurse managers and then synthesized the identified competencies with NMCI, the Army Leader Requirements model and the USU MEM Leadership Model to provide a standardized guideline for future military clinical nursing personnel management.

## 2 METHODS

A scoping review and a unifying framework development were undertaken focusing on competencies of military nurse leaders, in the way similar as that developed by Levac Colquhoun & O'Brien (2010). This methodology provides an overview of empirical and non‐empirical studies focusing on a particular topic and supports summarization of emerging evidence (Arksey & O'Malley, 2005; Levac et al., 2010). Additionally, a five‐step procedure, including formulating the research question; identifying relevant studies; selecting relevant studies; data charting; and collating, summarizing and reporting results, was taken (Levac et al., 2010).

### 2.1 Formulating the research question

The research question ‘what competencies of military nurse managers have been described?’ was formulated and used to guide this review.

### 2.2 Identifying relevant studies

‘Nurse manager’ in this review refers to managers at all levels including tactical/direct, operational/organisational and strategic level (VanFosson, 2012). Electronic searches were conducted to identify journal articles published in PubMed, CINAHL, EMBASE, PsycINFO, Cochrane Library and Chinese databases including CNKI and Wanfang up to November 2019. Grey literature was also searched in databases including Google Scholar. The following search theme was used: (a) military, or army, or air force, or navy, or warrior, or combat, or armed force, or defense; (b) nurse, or nursing; (c) head, or mid‐level, or manager, or leadership, or executive, or leader; (d) competence, or competency, or competencies.

### 2.3 Selecting relevant studies

Studies were included in the review if they (a) focused on competencies of military managers at different levels, including executive level, military nurse officer and head nurse; (b) were qualitative studies, quantitative studies, literature review or mixed‐method studies; (c) written in English or Chinese. Studies were excluded if they (a) were case studies, abstracts or citations; (b) were not specifically related to competencies of military managers; (c) publish with language other than English or Chinese. The selection of relevant studies was undertaken independently by two researchers.

**FIGURE 1:**
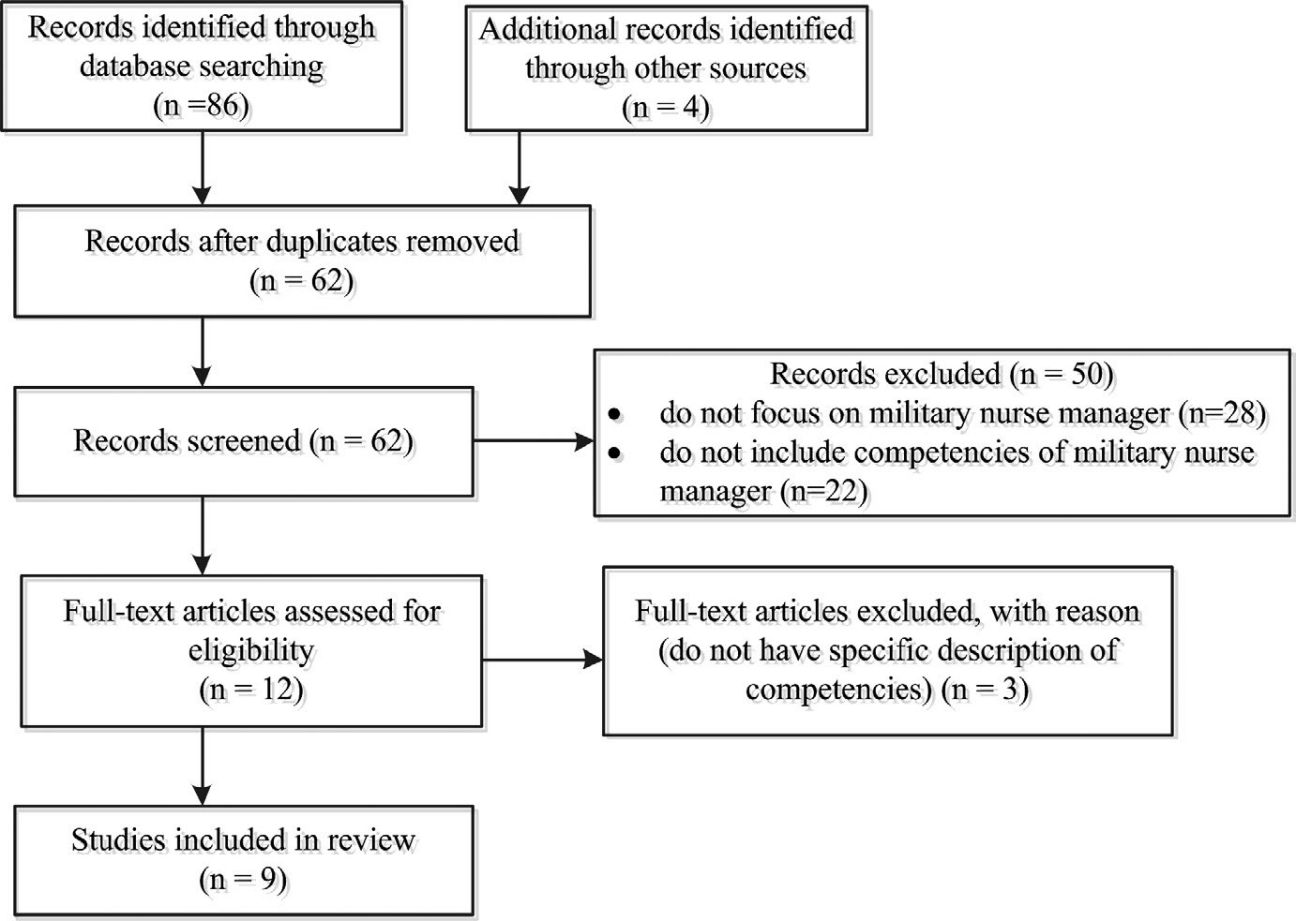
Flow chart of search process

A total of ninety studies (Figure 1) was yielded and then imported into EndNote X9 (Clarivate Analytics). After duplicates were removed, sixty‐two studies remained. These sixty‐two studies were assessed to determine whether they met the inclusion and exclusion criteria based on the title and abstract. Fifty studies failed to meet the inclusion criteria were excluded. The remaining twelve full‐text studies were retrieved and independently evaluated by two researchers. After careful examination based on inclusion and exclusion criteria, nine studies were remained and included in this review.

### 2.4 Data charting

Data from all the included studies were extracted and categorized into subgroups by the following headings: author, publish year, country, aim, design, type of nurse manager and all factors that could be identified as being related to competencies of military nurse managers (Table 1).

**TABLE 1:** Selected studies, aim, design, type of nurse manager, competencies derived from the studies and which domains of Chase Nurse Manager Competency Instrument the studies address: Chase Technical (T), Chase Human (H), Chase Conceptual (C), Chase Leadership (L) and Chase Financial Management (F)

Author (year) country	Aim	Design	Type of nurse manager	T	H	C	L	F	Competencies
Porter (2017) America	To develop executive‐level Army Public Health Nurse Competency and Evidence Based Toolkit for Leadership and Mentorship developmen	Qualitative and quantitative	Executive level	√	√	√	√	√	Analytical/assessment skills Policy development/programme planning skills and communication skills Cultural competency skills Community dimensions of practice skills Public health sciences skills Financial planning and management skills Leadership and systems thinking skills
Anderson (2016) America	To assess nurse manager competencies in a military hospital	Observational, descriptive, cross‐sectional approach	Head nurse	√	√		√		**Top 10 perceived competencies:** Effective leadership, decision‐making, problem‐solving, nursing practice standards(K), nursing practice standards(A), effective communication, time management, conflict resolution, infection control practices, effective staffing strategies
VanFosson (2012) America	To develop Adaptive Junior Leaders in the Army Nurse Corps	Literature review	Nursing officers	√	√	√	√		Foundational thinking Personal journey disciplines Systems thinking Succession planning Change management
Funari et al. (2011) America	To determine specific education and developmental experiences that will assist in developing ANC officers to become adaptive leaders	Literature review and qualitative study	Army Nurse Corps officers	√	√		√	√	Leadership Time management Budget management skills Complex multitasking Adaptability Soldier skills and developing a relationship with the operational officers Technical skills and clinical competence
Harris, (2007) America	To identify competencies and leadership characteristics of Army Adult Medical‐Surgical Critical Care Head Nurses	Qualitative study	Head nurse	√	√	√	√		Clinical expertise Know your staff Role model Communication and interpersonal skills Advocacy
Palarca, (2007). America	To achieve consensus among mid‐level Navy Nurse Corps officers about the relevant competencies and important skills, knowledge and abilities (SKAs) required for mid‐level leadership	Delphi technique	Mid‐level Navy Nurse Corps officers	√	√	√	√	√	Management Leadership Professional development Personal development Clinical growth and sustainment Deployment readiness and interoperability Regulatory guidelines
Dai (2010) China	To construct the competence model of employed head nurse in military hospital	Interview	Head nurse	√	√	√	√		Knowledge Nurse training Plan Vocational study Communication Detail/confidence Leading role
Ross (2010) US	To offer a brief look at patient care, deployment and leadership competency sets	Review	Nurse corps officers	√	√		√	√	Patient care competencies (ability to provide care for seriously injured combat or trauma patients, knowledge of battlefield, multitrauma injuries, ability to treat victims of chemical, biological or radiological terrorism) Deployment competencies ( knowledge of military mission, ability to interact according to military protocols, knowledge of military regulations, skills for patient movement from battlefield to stateside definitive care, knowledge of survival skills) Leadership competency ( supervision of a variety of enlisted personnel, ability to motivate personnel in high‐stress environments, personal skill in stress management; increased the demand for greater competency in finance management, information technology, data analysis, personnel management and organisational change)
Li (2007) China	To construct of competency model of head nurse in Chinese Army hospital	Behavioral Event Interview (BEI) questionnaire survey	Head nurse	√	√	√	√		Identification of job competence (including the dedication, passion for work, the development of subsidiary, coordination, innovation, adaptability, motivation, loyalty, team leadership, ability to learn and self‐ control, self‐confidence) Standard competence (including communication skills, combat awareness, service consciousness, attention to quality and order, integrity, supervisory ability, fairness and justice, social responsibility, correlated knowledge and skills)

### 2.5 Collating, summarizing and reporting the results

This procedure was divided into two stages. Stage one was to structure the competencies retrieved from the literature. When retrieving competencies, it was noted that NMCI was used as a tool in two studies. NMCI included five domains: (a) knowledge of health care environment (Chase Technical); (b) communication and relationship management (Chase Human); (c) professional (Chase Conceptual); (d) leadership (Chase Leadership); and (e) business skills and principles (Chase Financial Management). NMCI is a good framework for developing a comprehensive understanding of identified competencies from the included studies. So, five domains of NMCI were utilized to cross‐check the identified competencies of each study. Stage two was to outline the findings from stage one to develop a unifying framework, combined with NMCI, the Army Leader Requirements model and the USU MEM Leadership Model.

## 3 FINDINGS

### 3.1 Description of studies

A total of nine studies, published between 2007 and 2017, were included (Table 1).

These researches were undertaken in two countries: the United States (*n* = 7) and China (*n* = 2). The samples consisted of nurse managers from different organisational levels, including executive level (*n* = 1), military nurse officers (*n* = 4) and head nurses (*n* = 4). All the identified competencies were compared with the five domains of NMCI (Table 1). Nine studies mentioned competencies identified as Chase Technical, Chase Leadership and Chase Human. Six studies included competencies identified as Chase Conceptual, while four mentioned competencies identified as Chase Financial Management.

### 3.2 Comparison among military nursing managers of different levels

Anderson (2016) assessed the top 10 perceived competencies by using NMCI. These competencies matched three domains in the Chase competencies, including Chase Technical, Chase Human and Chase Leadership. Besides this study, eight other included studies also matched competencies in Chase Technical, Chase Human and Chase Leadership. Almost half (*n* = 4) of the eight studies that focused on the head nurse did not identified competencies of financial management, and the other four studies that identified finance relevant competencies with one study focusing on the nurse executive level, while the remaining three studies reviewed the military nurse officer level.

### 3.3 A framework of military nurse manager's competencies

Based on the competencies identified in stage one, a framework of military nurse manager's competencies was developed. The competencies identified in stage one were summarized into six domains of military nurse manager's competencies: (a) clinical expertise, (b) role model, (c) leadership competencies, (d) human competencies, (e) financial competencies and (f) deployment competencies (see Table 2).

**TABLE 2:** Conceptual framework of military nurse manager's competencies

Domain	Items
Clinical expertise	Clinical knowledge, clinical skills, assessment skills, nursing practice standards, infection control practice, evidence‐based practice and clinical diversity
Role model	Lead by example and display good characters (dedication, confidence, integrity, loyalty, passion for work, social responsibility)
Leadership competencies	Foundational thinking skills, personal journey disciplines, ability to use systems thinking, succession planning, change management and stress management
Human competencies	Communication and interpersonal skills (oral and written communication skills, interdisciplinary communication, communication at all levels, interpersonal skills, team building and positive work environment), organising (personnel management, staff development, professional development)
Financial competencies	Knowledge of basic business management practices (financial, supply and budget), formalize a strategic business plan, financial management skills, budget analysis and management skills, analytical ability and military medicine business practices
Deployment competencies	Combat casualty care competencies, military skills (knowledge of military mission and battlefield, ability to interact according to military protocols, knowledge of military regulations, survival skills) and military cultural competencies

#### 3.3.1 Clinical expertise

Clinical expertise includes clinical knowledge, clinical skills, assessment skills, nursing practice standards, infection control practice and evidence‐based practice, which provides the basis for completing complex multitasking (Anderson, 2016; Dai, 2010; Funari et al., 2011; Harris, 2007; Li, 2007; Porter, 2017; VanFosson, 2012). One unique aspect of clinical expertise in a military nursing context is clinical diversity. Military nurse managers should have multidisciplinary nursing knowledge and skills instead of focusing on one speciality. Clinical expertise can validate a nurse manager's leading role, help the manager to earn respect and influence others (Harris, 2007).

#### 3.3.2 Role model

A nurse leader is expected to be a role model for their staff in the way treating patients, family members and other health care team members in a kind and considerate manner (Harris, 2007). The term ‘role model’ refers to leading by example and displaying good character. Leading by example requires military nurse managers to exhibit good nursing practice at all times especially when treating multiple and serious‐wounded patients in austere environments (Dai, 2010; Harris, 2007). Their actions should be grounded in military values and professional ethics by displaying good characters such as dedication, confidence, integrity, loyalty, passion for work and social responsibility (Li, 2007).

#### 3.3.3 Leadership competencies

Leadership involves influencing all levels of personnel to accomplish a shared goal, and effective leadership can have a positive effect on patient outcome (Wong, Cummings, & Ducharme, 2013). Military nurse managers should have foundational thinking skills, personal journey disciplines, the ability to use systems thinking, succession planning, change management and stress management (Anderson, 2016; VanFosson, 2012). In detail, they should have the ability to lead a team, collaborate with individuals and organisations in developing a vision, motivating others, making decision, resolving conflicts, solving problems, managing change and stress, as well as being adaptable (Anderson, 2016; Dai, 2010; Funari et al., 2011; Harris, 2007; Li, 2007; Palarca, 2007; Porter, 2017; VanFosson, 2012).

#### 3.3.4 Human competencies

Human competencies refer to the ability to work effectively in a medical group and to exert a cooperative effort in the team (Katz, 1955). Human competencies include two key components: (a) communication and interpersonal skills and (b) organising. Communication and interpersonal skills can foster a positive clinical environment for patients and profession growth as well as facilitate teamwork and team success. These competencies consist of oral and written communication skills, interdisciplinary communication, communication at all levels, interpersonal skills, team building and maintaining a positive work environment (Anderson, 2016; Dai, 2010; Harris, 2007; Li, 2007; Palarca, 2007; Porter, 2017). Organising is the ability to plan activities and allocate resources including personnel management, staff development and professional development (Anderson, 2016; Dai, 2010; Harris, 2007; Li, 2007; Palarca, 2007).

#### 3.3.5 Financial competencies

In deployment to a conflict environment, military nurse managers might be provided with limited resources; thus, financial management is a required competency. Financial competencies include knowledge of basic business management practices (financial, supply and budget), formalizing a strategic business plan, financial management skills, budget analysis and management skills, analytical ability, and military medicine business practices (Funari et al., 2011; Palarca, 2007; Porter, 2017).

#### 3.3.6 Deployment competencies

Military nurse managers are trained to meet demanding military operational requirements during disaster relief, conflicts and peacekeeping missions. Deployment competencies include combat casualty care management, military skills and military cultural competencies (Li, 2007; Palarca, 2007; Porter, 2017; Ross, 2010). Combat casualty care management is necessary in caring for highly complex trauma patients such as victims of chemical, biological or radiological terrorism and seriously injured combatants (Ross, 2010). Military skills encompass knowledge of the military mission and battlefield, the ability to interact according to military protocols, knowledge of military regulations and survival skills (Palarca, 2007; Ross, 2010). Military cultural competencies include skills related to interfacing with patients of different cultural background and facilitating cooperation among three military services and civilian services (Ross, 2010).

## 4 DISCUSSION

Military nursing and civilian nursing have many similarities which are determined by the common goals to provide nursing care to patients and to promote health. However, the difference between the two is the mission of military nursing is to provide nursing care and leadership in both peacetime and during contingency operations (D'Angelo et al., 2019; Rivers & Gordon, 2017). Additionally, military nurses are required to work in a dynamic world of health care and to meet demanding military operational requirements (Anderson, 2016). Moreover, the military nursing context is determined by different working circumstances as the military nurses may work in military clinics, military base hospitals, tents, ambulances, airplanes, ships or alongside deployed troops. Furthermore, a transition from senior nurse to nurse manager might occur during deployment because of the military rank or clinical experience, indicating the importance of military nurse officers being cultivated to be managers from unit level to strategic level. This review aims to explore military nurse managers’ competencies to benefit the recruitment, training and management of military nurse leaders. Nine studies were included in this scoping review, and a unifying framework outlining six domains of military nurse managers’ competencies was developed.

Based on NMCI, the Army Leadership Requirements Model and the USU MEM Leadership Model, clinical expertise, role model, leadership competencies, human competencies, financial competencies and deployment competencies were identified. Army, Navy and Air Force services have unique nursing roles and demands in distinct competencies, and the above‐identified competencies of a military nurse manager were considered from a generic view instead of taking each military branch into account. Working in the military as a nurse manager requires them to sustain excellent performance in various military and health care environments.

Leadership is the ability to guide and manage, to make decisions under pressure or in dealing with unpredictable circumstances and to inspire other team members to follow (Brewer & Ryan‐Wenger, 2009). The Army Nursing Leader Capabilities Map clearly defined five key competencies of leadership, including foundational thinking, personal journey disciplines, systems thinking, succession planning and change management (VanFosson, 2012). Besides the above five competencies, stress management is also essential to leadership, especially when performing contingency operations (Anderson, 2016).

Foundational thinking skills refer to executing the vision, demonstrating evidence‐based decision‐making and developing internal standards, ethics and values while system thinking skills include understanding unit‐level processes and goals, as well as responding to divergent inputs to choose best clinical practices (VanFosson, 2012). Additionally, it is important for military nurse managers to understand the commander's missions, visions and to ground their actions of providing soldier‐centred care within military values and nursing ethics. However, dual loyalties can cause ethical problem which is the dilemma between providing nursing care and performing military missions (Draper & Jenkins, 2017; Lundberg et al., 2019). Based on the fact that leadership development is a lifetime career goal, from unit level to strategic level (Raimondo, Pierce, & Bruzek‐Kohler, 2008; Wilmoth & Shapiro, 2014), the personal journey disciplines enable nurse managers to seek self‐development, which includes seeking direct feedback and adjusting, applying new knowledge, setting initial personal, professional and career goals, as well as identifying positive role models (VanFosson, 2012). Besides focusing on personal development, military nurse managers should also put their effort into succession planning (Phillips, Evans, Tooley, & Shirey, 2018); in detail, they should motivate junior nurses, discover potentials of their staff and prepare themselves for the next leadership level (VanFosson, 2012). Personal journey disciplines and succession planning are the guarantees for the stable management of a nursing team from the unit level to the strategic level.

A role model competency is when a military nurse manager becomes a role model by committing themself to their duty, thereby encouraging their core team members to see how their roles contribute to the overall effectiveness of the team (Hughes, 2018). This is similar to the actions of Florence Nightingale who successfully led her team during the Crimean war (Stanley & Sherratt, 2010). Military nurse managers should lead by example in delivering nursing care, especially when faced with a dangerous work environment, or caring for infectious patients by displaying brave and professional actions grounded in military values and nursing ethics. This will help the managers to gain the loyalty and trust from their team members, as well as to motivate the team to sacrifice their time and energy for the benefit of others. When military nurse managers lead by example, they should also have clinical expertise with diverse clinical capabilities. These are determined by the fact that they are faced with a broad patient population that may be severely injured, suffering from shock, fractures or burns (Finnegan, Lauder, & McKenna, 2016; Rivers & Gordon, 2017), unlike civilian nurse managers who might focus on one specialty. Additionally, the dynamic changing world of health care requires military nurse managers to possess capabilities of change management and stress management, especially for military nurses who provide medical care in various high‐pressure environments (Anderson, 2016; Finnegan, Finnegan, & Thomas, 2014). Change management refers to identifying gaps, utilizing evidence‐based practice to initiate changes and adaptability to changes. When military nurse managers are deployed to combat zones or disaster rescue sites, they have to think quickly and clearly make life‐saving decisions. Under such circumstances, they need to take time to decompress from their pressure while relieving the stress of team members.

Military nurse managers should also possess human competencies, as they depend on their subordinates to achieve goals and to complete missions. It is essential for them to have good communication and interpersonal skills as well as organising abilities to motivate their subordinates and to assign tasks, especially when they are facing life‐threatening challenges when deployed in combat zones. For example, health care workers working in armed conflicts should know the importance of internal communication among their team and also consider the local context to prevent putting the team at risk (Baucom, 2017). Moreover, qualitative studies of deployment experiences constantly highlight the fact that military nurses felt unprepared to deliver nursing care in an austere environment (Conlon, Wiechula, & Garlick, 2019; Finnegan et al., 2016). This means military medical services should put more efforts to continuously teach, develop and prepare military nurses for future missions, either as a team member or as head of a medical team. To overcome these challenges, military nurse managers should be visionary and innovative, together with having competencies of communication skills, interpersonal skills, staff development, personnel management and professional development.

Financial competencies focus on cost–benefit analysis and financial resource monitoring. Military nurse officers might encounter a shortage of supplies in the midst of war, so supply management and improvising are important competencies of a battlefield nurse. The reason why three studies (Dai, 2010; Harris, 2007; Li, 2007) that targeted head nurses did not include any relevant competencies for financial management might be because head nurses working in hospital normally do not have much input on a budget; for example, some nurse managers in public hospitals rely on financial managers to give directions. Knowledge of health care budgets and costs enables nurse managers to adapt to changes in management and to improve nursing care. Therefore, military nurse managers shoulder responsibilities to manage resources during deployment especially in a combat zone, and they need to be educated in financial management by learning financial planning, financial monitoring, financial decision‐making and financial control (Naranjee, Ngxongo, & Sibiya, 2019).

Deployment competencies describe that the military medical force is expected to deliver expert medical care to support military operations, and military nurses should medically and physically fit for their role to be deployed on short notice (Kenward, Marshall, & Irvine, 2017). Deployment competencies, which are the basic of delivering patient care during deployment, refer to casualty management, military skills and military cultural competencies (Ross, 2010). Military nurses should be trained in combat casualty care to care for trauma patients in the battlefield. They should also have advanced capabilities for war wound care, as weapons of war evolve (Puri, 2017). Casualty management can be divided into three levels: first aid to wounded soldiers, quality medical treatment in a tactical environment and surgical capability in a military hospital (Andersson, Lundberg, Jonsson, Tingström, & Abrandt Dahlgren, 2017). Moreover, military nurses are challenged to have assessment skills, clinical skills, trauma care skills and critical care skills to save lives during military deployment (Agazio, 2010; Finnegan et al., 2014).

Deployment competencies can be gained through medical training, where the military nurse is trained as a professional nurse and as military personnel. Military training includes knowledge of military missions, protocols and regulations, as well as survival skills to be competent to adapt to various environments domestically or abroad. Military nurse managers should train subordinates to have a better understanding of the mission where they are deployed and maintain the physical fitness of junior officers. When deployed, military nurses are put in hostile environments with cultural challenges (Ross, 2010). Then, it is essential for military nurse managers to be able to care patients from diverse backgrounds and learn to cooperate with members from different institutions during deployment (Atuel & Castro, 2018; Ross, 2010). Besides, understanding diverse cultures is the cornerstone of patient care. It is also a substantial competence for military nurse managers to promote effective patient‐centred nursing practices among cross‐cultural contexts (Atuel & Castro, 2018; Meyer, Hall‐Clark, Hamaoka, & Peterson, 2015).

There are some limitations regarding this scoping review. This review summarized the competencies of military nurse managers identified from nine studies and developed a unifying framework, based on theoretical frameworks including NMCI, the Army Leader Requirements model and the USU MEM Leadership Model. We tried to expand search strategies to search more relevant published studies. However, due to the restricted distribution of relevant military files and language barrier, the resources we could get access were limited.

## 5 IMPLICATIONS FOR NURSING MANAGEMENT

Military nurse managers play a substantial role in ensuring nursing care quality in their units from disciplined subordinates who are loyal to the military service and nursing profession. They also are in charge of leading nursing team, cultivating junior military nurses, conveying military values and completing military missions. Moreover, the fluctuating nursing context from peacetime to different military missions poses challenge to the military nursing team. Military nurse managers are the core of this team; therefore, a comprehensive understanding of military nurse manager’ competencies and the unifying framework can provide directions to build a strong nursing team. This framework facilitates management of military nurse managers, including personnel recruitment, competency measurement and training protocol development. Additionally, this framework can also provide a basis for training civilian nurse managers to be better prepared for emergency public health event.

## 6 CONCLUSIONS

Existing knowledge of competencies of military nurse managers is limited, and the framework of competencies of military nurse managers developed in this review took the uniqueness of the military nursing context, as well as the common standard of each military branch and each manager level into consideration. Clinical expertise, role model, leadership competencies, human competencies, financial competencies and deployment competencies are key aspects for characterizing an excellent military nurse manager. A comprehensive understanding of this topic provides direction for future research work and nursing management.

## AUTHOR CONTRIBUTIONS

HM, TC, JF, LL and YL made substantial contributions to conception and design, or acquisition of data, or analysis and interpretation of data. HM, SZ, JT and LL involved in drafting the manuscript or revising it critically for important intellectual content. HM, TC, JF, SZ, LL, JT, LL and YL gave final approval of the version to be published and agreed to be accountable for all aspects of the work in ensuring that questions related to the accuracy or integrity of any part of the work are appropriately investigated and resolved. Each author should have participated sufficiently in the work to take public responsibility for appropriate portions of the content.
